# Expression of Retinal G Protein-Coupled Receptor, a Member of the Opsin Family, in Human Skin Cells and Its Mediation of the Cellular Functions of Keratinocytes

**DOI:** 10.3389/fcell.2022.787730

**Published:** 2022-04-04

**Authors:** Yangguang Gu, Yu Wang, Yinghua Lan, Jianglong Feng, Wen Zeng, Wei Zhang, Hongguang Lu

**Affiliations:** ^1^ Department of Dermatology, Affiliated Hospital of Guizhou Medical University, Guiyang, China; ^2^ Department of Dermatology and Venereology, Clinical College of Medicine, Guizhou Medical University, Guiyang, China

**Keywords:** RGR, proliferation, apoptosis, human skin, keratinocytes

## Abstract

**Background:** Photoreceptive proteins play critical physiological roles in human skin cells. The retinal G protein-coupled receptor (RGR) is a photoisomerase in the human retina, but its expression and cellular functions in human skin cells have not been reported.

**Objectives:** We aimed to detect RGR expression in various skin cells and evaluate its regulation of the cellular functions of keratinocytes.

**Methods:** The expression, distribution, and subcellular location of the RGR in normal human epidermal keratinocytes and cells with pathological conditions including psoriasis, seborrheic keratosis, and squamous cell carcinoma were determined using microscopic tools (immunohistochemical staining, immunofluorescence staining, and immunoelectron microscopy) and Western blotting (WB). The protein levels of the RGR in primary human melanocytes, keratinocytes, and fibroblasts isolated from the neonatal foreskin were measured by WB. The expression and subcellular localization of the RGR in these cells were detected by immunofluorescence staining under a fluorescence microscope and laser scanning confocal microscope. Additionally, the levels of RGR expression in normal keratinocytes exposed to ultraviolet (UV)-A or total ultraviolet radiation (UVR) in the presence or absence of all-trans-retinal were measured by WB. Furthermore, the effects of the RGR on human keratinocyte functions including proliferation, migration, and apoptosis were evaluated using the Cell Counting Kit 8, wound healing, and Transwell assays after reducing the RGR mRNA level in keratinocytes using small interfering RNA technology.

**Results:** The RGR was primarily located in the epidermal basal and spinous layers and skin appendages. Its expression increased in psoriatic lesions, seborrheic keratosis, and squamous cell carcinoma. Confocal microscopy showed that the RGR was located in the cell membrane and nucleus of keratinocytes, melanocytes, and fibroblasts. Keratinocytes had a higher expression of the RGR than melanocytes and fibroblasts, as well as nuclear expression, according to nuclear/cytoplasmic fractionation. Colloidal gold immunoelectron microscopy technology further confirmed that the RGR is mainly located in the nucleoplasm and mitochondria and is scattered in the cytoplasm and other organelles in the epidermal keratinocytes. Notably, RGR knockdown in keratinocytes led to the inhibition of cell proliferation and migration, augmenting cell apoptosis.

**Conclusions:** This study is the first to demonstrate the presence of RGR in the human skin. Our findings indicate that the RGR may play a critical role in the physiological function of epidermal keratinocytes.

## Introduction

Ultraviolet (UV) light plays a crucial role in human life (Holick, 2016). Humans can utilize energy from UV light *via* the eyes (Slominski et al., 2018). In the human retina, perception of the light signal results in photoisomerization, inducing conformational changes in opsin (OPN) (Maeda et al., 2010) and ultimately triggering the phototransduction cascade (Tsukamoto et al., 2005). In mice, M1-type intrinsically photosensitive retinal ganglion cells sense light signals to regulate hair follicle stem cell (HFSC) activation *via* melanopsin (Fan et al., 2018).

The photosensory system also exists in the skin (Moraes et al., 2021). The skin, as the largest organ of the human body and a defensive boundary, is constantly exposed to harmful external stimuli, including UV light, chemical toxins, and microorganisms (de Assis et al., 2020; Kusumoto et al., 2020). OPNs are photoreceptors that were recently discovered in the skin (Leung and Montell, 2017; Ozdeslik et al., 2019; Kusumoto et al., 2020; Lan et al., 2021; Wang et al., 2021) and belong to the G protein-coupled receptor (GPCR) family that mediates phototransduction in the human retina (Yau and Hardie, 2009). Human OPNs have been classified into nine subfamilies based on the chromosomal location and number of introns: rhodopsin, cone opsins (blue opsin, red opsin, and green opsin), encephalopsin, melanopsin, neuropsin, peropsin, and retinal G protein-coupled receptor (RGR) (Terakita, 2005). Among these OPNs, peropsin and RGR may serve as retinal photoisomerases (Terakita, 2005; Kiser et al., 2014; Morshedian et al., 2019) that catalyze the isomerization of the 11-cis-retinaldehyde chromophore of an opsin pigment to initiate visual perception. Under light stimulation, the RGR in the retinal pigment epithelium (RPE) can isomerize retinoids from the all-trans form into the 11-cis form (Jiang et al., 1993).

The RGR belongs to the retinal photoisomerase subfamily, which is one of the seven subfamilies of the OPN family (Terakita, 2005). The RGR gene has six introns and is different from other vertebrate visual and non-visual OPN genes in the molecular phylogenetic tree of the OPN family (Terakita, 2005). Although the RGR functions as an intracellular membrane-bound protein, similar to the other OPNs (Chen et al., 1996), it is mainly expressed in the RPE and glial Müller cells of the retina in mammals (Pandey et al., 1994). It may interact with the visual pigments colocalized in the outer cone segment (Zhang and Fong, 2018).

The RGR plays a role in the regeneration cycle of rhodopsin in mice (Chen et al., 2001). In humans, the RGR may contribute to the cone pigment regeneration (Morshedian et al., 2019). Notably, a two-point mutation in the human RGR gene has been found in patients with the photoreceptor degeneration (Morimura et al., 1999). These results suggest that the RGR is critical for the light-sensing function of the retina (Morimura et al., 1999; Morshedian et al., 2019). However, the absence of conserved (E/D) R (Y/W/F) and NPxxY(x)5,6F motifs in the structure of the RGR makes it different from other OPNs (Fritze et al., 2003). Thus, the RGR is not considered a signaling molecule (Hao and Fong, 1999). Additionally, mutations in the RGR gene can be complicated in patients with dominantly inherited peripapillary choroidal atrophy (c.824dupG, p. I276Nfs*77) (Kochounian et al., 2016). The overexpression of RGR-d (an exon-skipping splice variant of RGR-opsin) (NP_001012740) in the ARPE-19 and COS-7 cell lines causes cell growth inhibition (Bao et al., 2021). Although many reports have investigated RGR functions, these functions remain incompletely elucidated. Our group and others have demonstrated that some OPNs expressed in the eyes are found in the human skin (Regazzetti et al., 2018; Olinski et al., 2020; Lan et al., 2021). However, whether the RGR is expressed in the human skin and, if so, what are its cellular functions remain unknown.

Here, we aimed to examine the expression and subcellular localization of the RGR in skin tissues and their cells, including melanocytes, keratinocytes, and fibroblasts. We further explored RGR expression in skin lesions associated with the keratinocyte-proliferative diseases such as psoriasis, seborrheic keratosis, and squamous cell carcinoma. We also aimed to elucidate the relationship between the RGR expression and proliferation of keratinocytes.

## Materials and Methods

### Cell Culture

Human primary melanocytes, human primary keratinocytes, and human primary fibroblasts were derived from the neonatal foreskin of children aged 3–12 years using a two-step enzyme digestion method at the Pediatric Surgery Departments of the Affiliated Hospital of Guizhou Medical University. Non-tumorigenic human keratinocytes (HaCaT cell line) were purchased from the Kunming Cell Bank of Type Culture Collection of Chinese Academy of Sciences (Kunming, China) (cat. No. KCB200442YJ). Melanocytes were cultured in complete Medium 254 (cat. No. M254500; Invitrogen Cascade Biologics, America) supplemented with Human Melanocyte Growth Supplement-2 (cat. No. S0165; HMGS2; Gibco, United States), 2 mM L-glutamine (cat. No. 1051024; Gibco, United States), and 100 units/mL of penicillin–streptomycin (cat. No. P1400; Solarbio, China) and used at their third passage. Keratinocytes were grown in EpiLife^®^ medium (cat. No. MEPI500CA; Invitrogen Cascade Biologics, America) supplemented with Human Keratinocyte Growth Supplement (cat. No. S0015; HKGS; Invitrogen Cascade Biologics, America), 2 mM l-glutamine (cat. No. 1051024; Gibco, United States), and 100 units/mL of penicillin–streptomycin (cat. No. P1400; Solarbio, China) and used at their third passage. Fibroblasts and HaCaT cells were grown in Dulbecco’s modified Eagle’s medium (cat. No. 0030034DJ; Gibco, United States) supplemented with 10% fetal bovine serum (FBS) (cat. No. FBSSA500-S; FBS; AUSGeneX) and 100 units/mL of penicillin–streptomycin (cat. No. P1400; Solarbio, China). The aforementioned cells were cultured at 37°C in a humidified incubator (Forma, United States) with 5% CO_2_. The study was approved by the Ethics Committee of the Affiliated Hospital of Guizhou Medical University.

### Clinical Samples

A total of six healthy subjects who had undergone plastic and aesthetic surgery were recruited from the Department of Plastic Surgery, Affiliated Hospital of Guizhou Medical University, between January 2019 and July 2021. Normal skin tissues from paired, exposed, and unexposed areas were collected. Skin tissues of the lesion and matched adjacent normal areas were collected from six patients with psoriasis, 6 with seborrheic keratosis, and 6 with squamous cell carcinoma at the Dermatology Department, Affiliated Hospital of Guizhou Medical University, between January 2019 and July 2021. The diagnoses of all the patients were based on clinical examinations and skin biopsy. We obtained a written informed consent from each participant. Clinical skin specimens were obtained with the approval of the Ethics Committee of the Affiliated Hospital of Guizhou Medical University, and all the analyses of human materials were in full compliance with our institutional guidelines.

### Western Blot Analysis

The cells, the entire skin tissues of lesions, paired adjacent normal skin tissues (PANST), and normal skin tissues were lysed in RIPA lysis buffer (cat. No. R0010; Beijing Solarbio Science and Technology Co., Ltd., Beijing, China) supplemented with 1 mM PMSF (cat. No. R0010; Beijing Solarbio Science & Technology Co., Ltd., Beijing, China). The nuclear and cytoplasmic proteins of primary human keratinocytes were extracted using a Nuclear and Cytoplasmic Protein Extraction Kit (cat. No. P0027; Beyotime Institute of Biotechnology, Nantong, China) according to the manufacturer’s protocols. The protein contents were determined using the BCA method (cat. No. PC0020; Beijing Solarbio Science and Technology Co., Ltd., Beijing, China). The protein samples were mixed with sample loading buffer (cat. No. P0015A; Beyotime Institute of Biotechnology, Shanghai, China) and denatured for 10 min at 100°C. Next, 40 µg of protein was separated by 5% to 10% sodium dodecyl sulfate–polyacrylamide gel electrophoresis (SDS–PAGE) and transferred onto polyvinylidene difluoride (PVDF) membranes (EMD Millipore, Billerica, MA, United States) for 90 min at 250 mA. The membranes were subsequently blocked with 5% non-fat milk for 2 h at room temperature and incubated with primary antibody overnight at 4°C. For WB, rabbit anti-human RGR monoclonal antibody (cat. no. PA5-100993; 1:1,000; Invitrogen Biosciences, Beijing, China) was used as the primary antibody. The following primary antibodies were used as internal controls: mouse anti-human lamin-B monoclonal antibody (cat. no. DF7356; 1:1,000; Affinity Biosciences, Beijing, China), mouse anti-human *β*-tubulin monoclonal antibody (cat. no. T0023; 1:10,000; Affinity Biosciences, Beijing, China), and mouse anti-human glyceraldehyde 3-phosphate dehydrogenase (GAPDH) monoclonal antibody (cat. no. DF7967; 1:10,000; Affinity Biosciences, Beijing, China). The membranes were incubated with horseradish peroxidase-labeled goat anti-rabbit IgG (cat. no. ab6721; 1:10,000; Abcam, United States) or horseradish peroxidase-labeled goat anti-mouse IgG (cat. no. ab6789; 1:10,000; Abcam, United States) for 45 min at room temperature. Finally, the membranes were visualized using a BeyoECL Plus kit (P0018S; Beyotime Institute of Biotechnology, Shanghai, China) with the Bio Imaging system (Bio–Rad Laboratories, Inc.). The RGR expression levels were measured using the Bio Imaging system (Bio–Rad Laboratories, Inc.) after normalization to *β*-tubulin, Lamin-B or GAPDH.

### Histological and Immunochemical Assays

Six fresh normal skin tissues, including paired exposed and unexposed areas, were available. According to the streptavidin peroxidase-conjugated method, the skin tissue samples were fixed in 10% formalin solution for 6–12 h at room temperature and embedded in paraffin. Formalin-fixed sections, with 4 μm thickness, were stained with hematoxylin and eosin for further study. The sections were incubated with rabbit anti-human RGR monoclonal antibody (cat. no. PA5-100993; 1:200; Invitrogen Biosciences) at 4°C overnight, incubated with horseradish peroxidase-conjugated IgG goat anti-rabbit secondary antibody (1:150; DAKO, Glostrup, Denmark) for 30 min, and finally visualized with 3-amino-9-ethylcarbazole (DAKO). The immunohistochemical scores were determined in a blinded manner under a light microscope. The staining intensity and percentage of stained keratinocytes in five fields of sections were qualitatively scored using the scores of 3+ (strong), 2+ (moderate), 1+ (weak), and 0 (negative) staining for each case, as described previously. The overall RGR expression score was obtained by summing the staining intensities and corresponding percentages according to the calculation formula (3 × x% + 2 × x% + 1 ×x% = total score) (Toyama et al., 2019).

### Immunofluorescence

#### Tissue Immunofluorescence

The skin samples were fixed in 4% paraformaldehyde and embedded in paraffin. Then 5–8 μm thick sections were dried at 37 C, deparaffinized in xylene, and hydrated in a graded series of ethanol. Antigens were retrieved by microwave heat treatment. After blocking with 2% BSA (cat. no. ZLI-9022; ZSGB-BIO Biosciences, Beijing, China) for 1 h at room temperature, the slides were incubated with anti-RGR antibodies (cat. no. ABP56042; 1:200; Abbkine Biosciences, Beijing, China) overnight at 4°C. Next, the sections were incubated with a fluorescence-labeled secondary antibody for 50 min at room temperature. The sample was visualized under a Cell Observer, Living Cells (Zeiss, Germany).

### Colloidal Gold Immunoelectron Microscopy (IEM)

The skin tissue was cut into thin slices of approximately 50–70 nm and loaded onto a 200- to 300-mesh nickel grid. After the treatment with 1% H_2_O_2_ for 10 min to 1 h and three 10-min washes with phosphate-buffered saline (PBS), thin slices were incubated with a normal sheep serum (1:100) at room temperature for 60 min. Following a brief wash with PBS, the sections were treated with 2% fish gelatine–PBS for 30 min, incubated with anti-RGR antibody (cat. no. PA5-100993; 1:1,000; Invitrogen Biosciences) overnight at 4°C, and then washed with PBS. Next, the sections were incubated with rabbit anti-mouse IgG (1:1,000) for 30 min at room temperature, followed by 30-min incubation with a protein A-gold probe (15 nm gold particles). After washing with PBS, the sections were fixed with 1% glutaraldehyde in PBS for 10 min, washed with distilled water, and then contrasted with uranyl acetate and lead citrate. Finally, the sections were examined under a Hitachi H7650 electron microscope (Hitachi, Tokyo, Japan) at an acceleration voltage of 80 kV. For the negative control, the sections were incubated with the control antibody.

### Cellular Immunofluorescence

Keratinocytes were seeded on coverslips at a density of 1.2×10^4^ cells/well and then cultured at 37°C with 5% CO_2_ for 24 h. The cells were fixed with 95% ethanol at room temperature for 10 min, followed by blocking with 10% FBS for 30 min at 37°C. After washing with PBS, the cells were incubated with mouse anti-RGR monoclonal antibody (cat. no. ABP56042; 1:200; Abbkine Biosciences) overnight at 4°C. Following another wash with PBS, the cells were incubated with goat anti-mouse IgG FITC-labeled fluorescent antibody (cat. no. MD6640-100; 1:50; MDL, Beijing, China) for 45 min in the dark at room temperature. Finally, the cells were stained with 4′,6-diamidino-2-phenylindole (DAPI) (cat. no. C0065; Solarbio, Beijing, China) for 10 min at room temperature and imaged by using a confocal microscope (Zeiss, Oberkochen, Germany).

### Ultraviolet Radiation

Primary human keratinocytes were seeded on coverslips at a density of 1.2×10^4^ cells/well. The light source used was a UV light therapy unit (Sigma High-tech Co., Ltd., Shanghai, China) that emitted a spectrum from 320 to 400 nm for total UVA and from 280 to 400 nm for total UV radiation (UVR). The UV dose was measured by using a UV radiometer (Sigma High-tech Co., Ltd., Shanghai, China). The keratinocytes were irradiated with 1.5 J/cm^2^ UVR or 3 J/cm^2^ UVA in the presence or absence of all-trans-retinal. After culturing for 24 h, the cells were collected for further experiments.

### Cell Transfection

Silencing of the RGR in primary human keratinocytes or HaCaT cell lines was performed using RGR small interfering RNA (siRNA), following the manufacturer’s protocol. The RGR-specific siRNA (5′-AUG​CCA​UCC​UGU​AUC​UAU​ATT-3′) and negative-control siRNA (5′-UAU​AGA​UAC​AGG​AUG​GCA​UTT-3′) were purchased from Tran Sheep Bio-Tech Co. Ltd. (Shanghai, China). The cells were seeded in 6-well plates at a concentration of 10^4^ cells/well and reached 30% confluence. Next, the cells were treated with 40 nM RGR siRNA and 5 µl of Lipofectamine 2000 (cat. no. 2097561, Invitrogen, United States) for 48 h. Transfection reactions were performed in the serum-free Opti-MEM (cat. no. 31985070; Gibco, United States). After 20 min of treatment, complete culture medium was added to the cells. The silencing effect of RGR was assessed 48 h after the siRNA transfection by Western blotting.

### Cell Viability Assay

The cell viability of primary human keratinocytes or HaCaT cell lines was determined using the Cell Counting Kit-8 (CCK-8) assay (GLPBIO, United States) according to the manufacturer’s instructions. Briefly, the cells were incubated overnight in 96-well culture plates at a concentration of 1×10^3^ cells/well and were grown in complete medium for 12–16 h. When 30% confluence was reached, the cells were treated with a mixture of 40 nM RGR siRNA and 5 µl of Lipofectamine 2000 for 48 h, as mentioned previously. Next, 10 µl of CCK-8 solution was added to each well, and the cells were incubated at 37°C for another 2 h. The absorbance at 450 nm was then measured by using a microplate reader.

### Wound Healing Assay

The cells were incubated in a 96-well plate at a density of 1×10^3^ cells/well. When they reached confluence in the monolayer, straight lines were drawn by scraping the confluent cells with a 10-µl pipette tip. Following washing with PBS, the cells were incubated in serum-free medium for 48 h. Photographs of the scratch were taken in the same position at 0, 24, and 48 h. Cell migration was analyzed by measuring the cell-covered area using ImageJ 6.0 software (National Institutes of Health, United States).

### Transwell Migration Assay

For the Transwell migration assay, a chamber assay was performed using an 8 µm pore size (Corning, Lowell, MA, United States). A total of 1×10^4^ cells suspended in 200 μl of serum-free medium were added to the upper chamber, while 400 μl of the complete medium containing 30% FBS was added to the bottom of each chamber. After 48 h of incubation, the cells that passed through the membrane were fixed with 4% paraformaldehyde, stained with 0.1% crystal violet (cat. G1063; Solarbio, China), and imaged at ×100 magnification in five random fields. The cells that passed through the membrane were counted using ImageJ 6.0 software (National Institutes of Health, United States).

### Flow Cytometry Analysis

To estimate the level of apoptotic cell death following RGR silencing, cells were dually stained with Annexin V-FITC/PI mix and analyzed using a BD FACSCalibur Flow Cytometer (BD Biosciences, San Jose, CA, United States). Following the aforementioned steps of cell transfection, the cells were seeded into 6-well plates at a concentration of 10^4^ cells/well for 12–14 h. When 30% confluence was reached, the cells were treated with a mixture of 40 nM RGR siRNA and 5 µl of Lipofectamine 2000 for 48 h, as previously mentioned. Next, 400 μl of annexin buffer containing 5 μl of Annexin V-FITC (cat. no. A005-3; 7sea Biotechnology, Shanghai, China) was added to resuspend the cells, which were then incubated for 15 min at room temperature in the dark. After 10 μl of PI was added to the buffer, the percentage of apoptotic cells was determined by flow cytometry with Cell Quest software. The cells treated with a mixture of 40 nM RGR siRNA and 5 µl of Lipofectamine 2000 for 48 h were also processed for flow cytometry to analyze the cell cycle distribution. The cells were fixed in 70% ice-cold ethanol overnight, incubated with 1 mg/ml of RNase A at 37°C for 30 min, and stained with PI for 1 h in the dark (cat. no. C001-507; 7sea Biotechnology, Shanghai, China). The cell cycle distribution was analyzed by flow cytometry (BD Biosciences, San Jose, CA, United States).

### Statistical Analysis

All the experiments were performed at least three times independently. Statistical analyses were performed using GraphPad Prism 8.0 software (GraphPad Software, San Diego, CA). Significant differences between the groups were determined using Student’s t test and one-way ANOVA with Tukey’s post hoc test. *p* < 0.05 was taken as the measure of statistically significant differences.

## Results

### RGR Is Expressed in Human Melanocytes, Keratinocytes, and Fibroblasts

**FIGURE 1:**
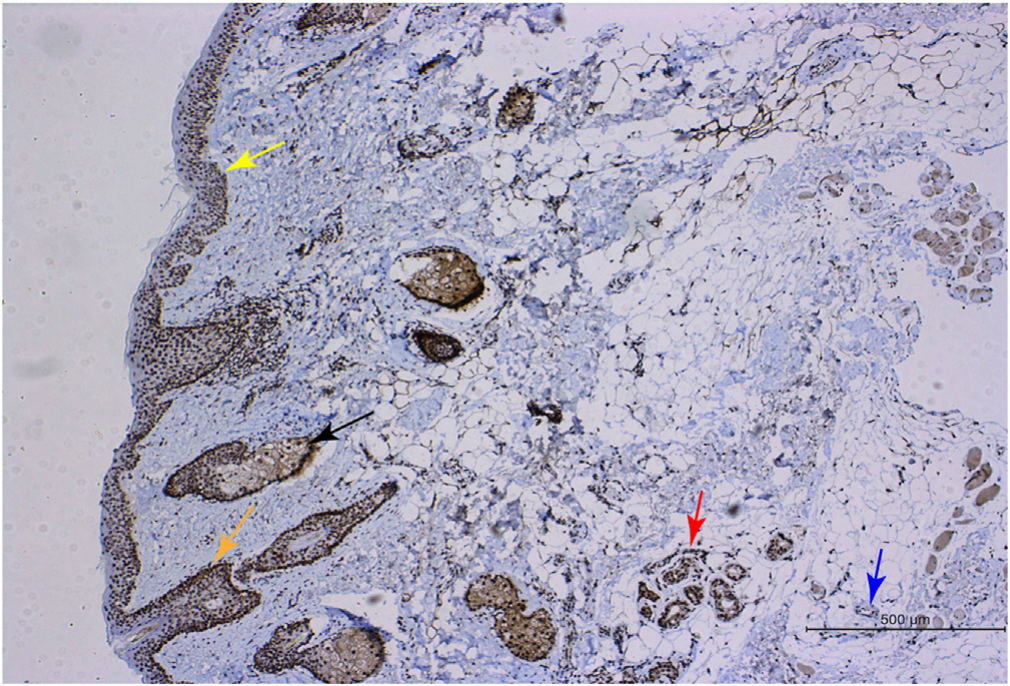
RGR expression in human skin tissue. Immunohistochemical methods were used to show positive RGR staining in the epithelial layer (yellow arrow), sebaceous gland (black arrow), hair follicle (orange arrow), sweat gland (red arrow), and blood vessel (blue arrow) of skin tissue. Scale bar = 500 μm.

**FIGURE 2:**
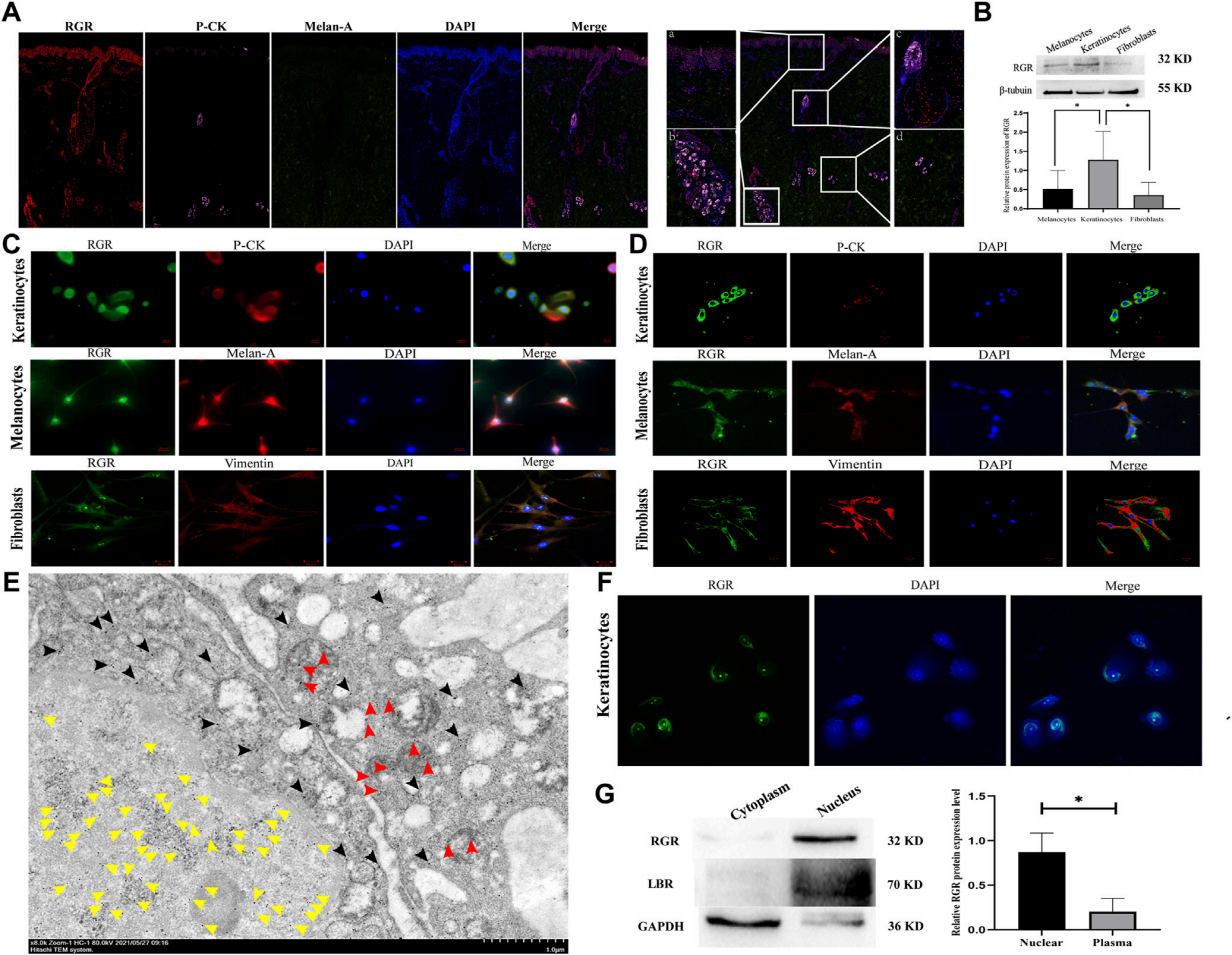
RGR expression level and subcellular localization in human skin cells. **(A)** In normal human skin tissue, coimmunostaining of RGR (red) with the melanocyte marker melan-A (green) and keratinocyte marker P-CK (pink) is shown. Scale bar = 20 μm. The results showed positive staining of the RGR in the basal layer of the skin, large sudoriferous glands, hair follicles, and eccrine glands. Scale bar = 200 μm. **(B)** Representative expression of RGR and *β*-tubulin (loading control) in melanocytes, keratinocytes, and fibroblasts, as measured by the Western blot assay (anti-RGR antibody: a band of ∼32 kDa; *β*-tubulin antibody: a band of ∼55 kDa). The relative protein levels of RGR and *β*-tubulin were measured by ImageJ software (n = 3 independent experiments). **(C)** In cultured cells, the colocalization of the RGR (green) and melan-A (red) in melanocytes, colocalization of RGR (green) and CK-pan (red) in keratinocytes, and colocalization of RGR (green) and vimentin (red) in fibroblasts were detected by immunofluorescence double staining. Scale bar = 20 μm. **(D)** Localization and expression of the RGR (green) in human skin cells (melanocytes, keratinocytes, and fibroblasts) were observed by a confocal microscope. Scale bar = 20 μm. **(E)** IEM staining of the normal skin tissue. Representative protein A-gold probe represents RGR (RGR is mainly located in the nucleoplasm (yellow arrow) and mitochondria (red arrow) and scattered in the cytoplasm and other organelles (black arrow)). Scale bar = 1 μm. **(F)** Representative expression of RGR (green) in the membrane and nucleus of human keratinocytes was observed by confocal microscopy. Scale bar = 20 μm. **(G)** Representative differential expression of RGR, lamin B (nuclear control antibody), and GAPDH (loading control) in the human keratinocyte membrane, and the nucleus was analyzed by Western blotting. The relative protein levels were quantified using ImageJ software (n = 3 independent experiments). *p < 0.05, **p < 0.01, ***p < 0.001 vs the control group.

The RGR is mainly expressed in the RPE and glial Müller cells of the retina in mammals and may interact with the visual pigments colocalized in the outer cone segment (Kiser and Palczewski, 2021). We first investigated the expression and distribution of the RGR in human skin tissue. When the RGR was detected in formalin-fixed and paraffin-embedded normal skin tissues by immunohistochemistry, the results showed positive staining of the RGR in the basal layer of skin, sebaceous gland, hair follicles, sweat glands, and blood vessels (Figure 1). To further clarify RGR expression, we used immunofluorescence and multiplex immunofluorescence staining and coimmunostaining of the RGR with the melanocyte marker melan-A and keratinocyte marker P-KC. RGR was mainly expressed in the nucleus of the epidermal basal cell layer and spinous layer (Figure 2A). Additionally, RGR staining was positive in the skin sebaceous gland, and the vascular RGR immunofluorescence was observed (Figure 2A). To further confirm the RGR expression in these cells, we isolated and cultivated the normal human keratinocytes, melanocytes, and fibroblasts from the foreskin of children. The protein levels of RGR were detected by WB, which showed that RGR was expressed in the keratinocytes, melanocytes, and fibroblasts (Figure 2B). Interestingly, the protein level of RGR in the keratinocytes was higher than that in the other cell types (Figure 2B).

Subcellular localization is critical for the cellular function of a protein. We detected the location of RGR in the normal human keratinocytes, melanocytes, and fibroblasts by observing the colocalization of immunofluorescence staining for RGR with that of melan-A, pan-CK, and vimentin. The RGR showed strong staining in the cell membrane of keratinocytes, melanocytes, and fibroblasts under an immunofluorescence microscope and a confocal microscope (Figure 2C). In keratinocytes, melanocytes, and fibroblasts, we also detected dispersed expression in the nucleus (Figure 2D). To further determine the subcellular localization of the RGR, we used IEM technology to detect the RGR in normal human skin tissues. The highly magnified view of the RGR revealed that it was mainly located in the nucleoplasm (yellow arrow) and mitochondria (red arrow), scattered in the cytoplasm (black arrow), and other organelles in the spinous cell layer of keratinocytes (Figure 2E). Additionally, the fluorescence intensity of the RGR in keratinocyte nuclei was higher than that in membranes (Figure 2F). Next, nuclear pulp separation was used to evaluate whether the RGR cell membrane and nuclear expression levels differed. RGR expression in the nucleus was similar to that of the nuclear control antibody lamb and was significantly higher than that in the cytoplasm (Figure 2G). These results led us to perform further experiments on the RGR function in the skin cells.

### UVA and UVR May Not Induce RGR Expression in Human Keratinocytes Under Physiological Conditions

**FIGURE 3:**
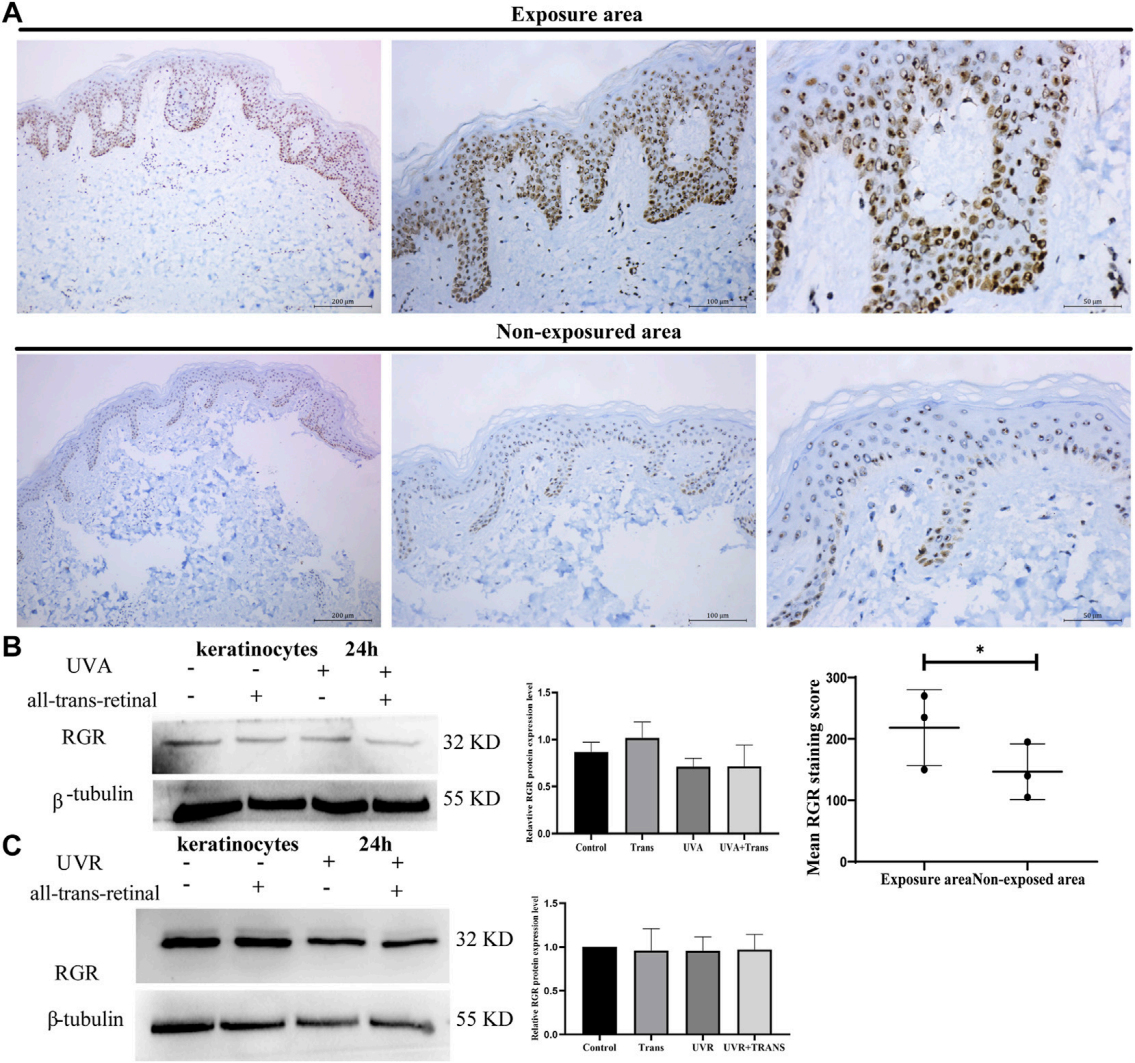
UVA and UVR may not induce RGR expression in human keratinocytes. **(A)** The differential RGR expression in the exposed and unexposed skin tissues was analyzed by immunohistochemistry. The RGR was highly expressed in the epithelial layer of light-exposed sites, particularly in the basal cell layer and spinous cell layer of the epidermis and cells of the skin appendages. Exposed area vs unexposed area, *p < 0.05. Scale bar = 100 μm (right), 50 μm (middle), 20 μm (left). **(B)** Keratinocytes with all-trans-retinal were irradiated with 3 J/cm^2^ UVA. Representative expression of RGR and *β*-tubulin (loading control) by Western blot analysis. The relative protein levels of RGR and *β*-tubulin were measured using ImageJ software (n = 3 independent experiments). **(C)** Keratinocytes with all-trans-retinal were irradiated with 1.5 J/cm^2^ UVR. Representative expression of RGR and *β*-tubulin (loading control) was detected by Western blot analysis. The relative protein levels of RGR and *β*-tubulin were measured using ImageJ software (n = 3 independent experiments).

We next explored the potential roles of the RGR in skin cells. Previous studies have demonstrated that the RGR may act as photoisomerase in the visual system (Morshedian et al., 2019), but whether the RGR can sense light in the skin as in the retina remains unclear. Immunohistochemical staining of the sections of normal skin tissue also showed a higher expression level of the RGR in the epithelial layer of exposed skin areas, particularly in the basal and spinous cell layers of the epidermis and cells of the skin appendages (Figure 3A). We wondered whether these expression differences in the RGR were related to light. The RGR may specifically bind to all-trans-retinal *via* its lysine residue at position 255 (Choi et al., 2021). The combination of the RGR and all-trans-retinal shows different UV and visible light absorption than the RGR alone (Hao and Fong, 1996). The RGR absorption in the blue and near-UV regions of light depends on pH. When the pH ranges from 6.5 to 4.0, the absorbance by the RGR of blue light increases noticeably. The large peak of the RGR in the UV region is unaffected by pH (Hao and Fong, 1996). Based on these results, keratinocytes were exposed to UVA (3 J/cm^2^) or UVR (1.5 J/cm^2^) at physiologically relevant doses in the presence or absence of all-trans-retinal (Wicks et al., 2011). Interestingly, we found that the protein level of the RGR in keratinocytes did not change after 24 h of exposure to UVA or UVR (Figures 3B,C). Additionally, after we added all-trans-retinal and irradiated the cells with UVA or UVR, RGR expression in the all-trans-retinal-UVA group and all-trans-retinal-UVR group did not change compared with that in the control group (Figures 3B,C).

### Is Overexpression of RGR Associated With Proliferative Diseases and Skin Tumors?

**FIGURE 4:**
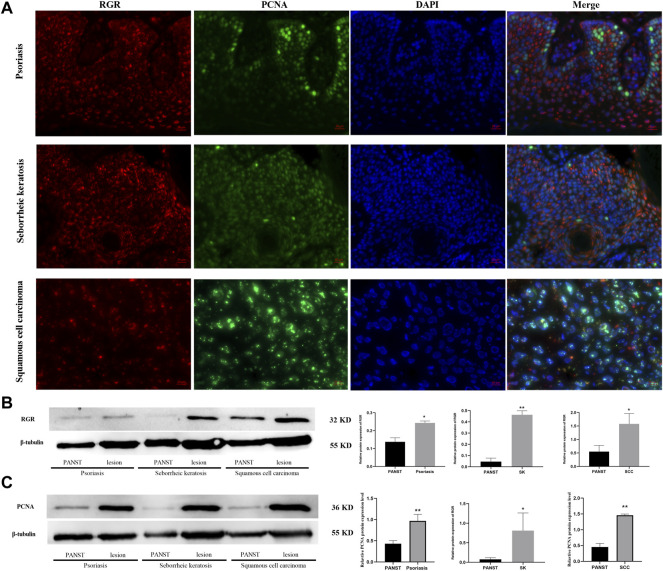
RGR is highly expressed in proliferative diseases and tumor tissues. **(A)** RGR expression (red) is colocalized with the proliferative marker PCNA (green) in psoriasis, seborrheic keratosis, and squamous cell carcinoma. Scale bar = 20 μm. **(B)** Representative expression of RGR and *β*-tubulin (loading control) in lesion areas and non-lesion areas was detected by WB analysis. The relative protein levels of RGR and *β*-tubulin were measured using ImageJ software (n = 3 independent experiments). *p < 0.05. **(C)** Representative expression of PCNA and *β*-tubulin (loading control) was detected by WB analysis. The relative protein levels of RGR and *β*-tubulin were measured using ImageJ software (n = 3 independent experiments). **p < 0.01.

Abnormal RGR expression is related to cell development and even disease progression (Morimura et al., 1999; Bao et al., 2021). We further explored RGR expression in some skin tissues from pathological conditions, including benign proliferative diseases and skin tumor tissues. The fluorescence intensity of the RGR in some benign and malignant proliferative disease tissues (seborrheic keratosis, psoriasis, and squamous cell carcinoma) lesions was higher than that in the adjacent lesions (Figure 4A). This finding was further confirmed by Western blot analysis, which showed that RGR expression in the lesion area was significantly higher than that in the lesion-adjacent area (Figure 4B). At the protein level, PCNA expression in the lesioned tissues was also significantly higher than that in the adjacent tissues (Figure 4C). These observations suggested that the RGR may be associated with certain skin diseases, but detailed mechanistic studies are needed.

### RNAi Knockdown of RGR in Keratinocytes Leads to Reduced Proliferation, Migration, and Apoptosis

**FIGURE 5:**
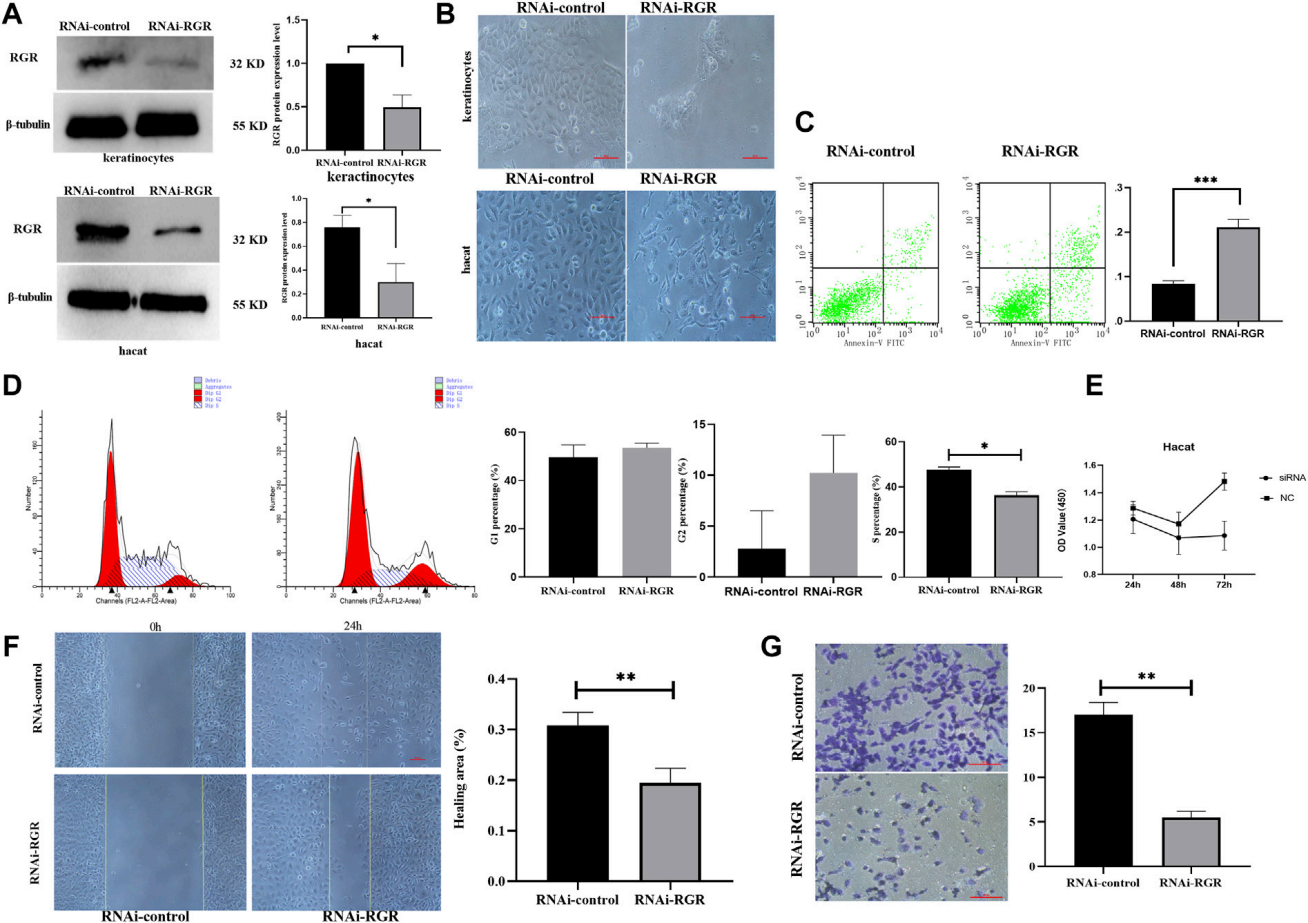
RGR regulates the proliferation, migration, and apoptosis of keratinocytes. **(A)** Representative keratinocyte defects in RGR (with small siRNA) and *β*-tubulin (loading control) were detected by WB analysis. The relative protein levels of RGR and *β*-tubulin were measured using ImageJ software (n = 3 independent experiments). *p < 0.05, **p < 0.01, ***p < 0.001. **(B)** Representative images of cell fragmentation and cell contraction observed under a light microscope in the si-RGR transfection group. No significant morphological change was detected in the si-control group. Scale bar = 20 μm *p < 0.05, **p < 0.01, ***p < 0.001 vs the control group. **(C)** Flow cytometry with annexin V FITC/PI double staining was used to determine apoptotic cells. Following RGR knockdown, a significant decrease in apoptosis was detected in the keratinocytes transfected with si-RGR compared with the control. *p < 0.05, **p < 0.01, ***p < 0.001 vs the control group. **(D)** Flow cytometry was used to detect the cell cycle after RGR knockdown in keratinocytes. Following RGR knockdown, a significant decrease in the S phase at 48 h was detected in keratinocytes transfected with si-RGR compared with the control. *p < 0.05 vs the control group. **(E)** RGR in keratinocytes was inhibited *via* Si-RGR, and cell viability was measured after 48 h. **(F)** Representative images from wound healing experiments showed low expression of the RGR and decreased mobility relative to controls. Scale bar = 50 μm *p < 0.05, **p < 0.01, ***p < 0.001 vs the control group. **(G)** Transwell assays showed that knocking down RGR reduced the invasion by keratinocytes. Scale bars = 50 μm.

To investigate whether RGR can mediate skin functions in keratinocytes, we knocked down RGR in keratinocytes using siRNA technology. WB showed that the protein level of RGR in the keratinocytes treated with RGR siRNA was reduced by more than 60% compared with that in the control group (Figure 5A). Surprisingly, significant morphological changes in cells, including cell shrinkage and cell fragmentation, were observed in keratinocytes transfected with si-RGR (Figure 5B). To determine the possible mechanism of such changes in keratinocytes, the annexin V/PI staining assay (Wang et al., 2020) was performed to detect apoptotic and viable keratinocytes treated with RGR siRNA. A portion of cells (20%) were apoptotic in keratinocytes transfected with si-RGR, while only 8% were apoptotic in keratinocytes transfected with RNAi-control (*p* < 0.05) (Figure 5C). These data suggest that keratinocyte apoptosis occurred after downregulating RGR expression. Next, we analyzed the changes in the cell cycle of si-RGR keratinocytes, which exhibited cell cycle arrest at the G1/S phase at 48 h (Figure 5D). Cell viability was lower in the group treated with si-RGR than in the si-control group (Figure 5E). Additionally, wound healing and Transwell assays were used to analyze the cell migration ability after downregulating RGR expression in keratinocytes with the siRNA (Figures 5F,G). Interestingly, the cell migration ability in the group treated with si-RGR was significantly lower than that of the control group (Figure 5G). These results suggested that RGR may play a crucial role in the physiological function of keratinocytes.

## Discussion

This work describes the expression, distribution, and subcellular location of the RGR in normal human skin tissues, pathological skin tissues, and normal human skin cells (keratinocytes, melanocytes, and fibroblasts). Our work demonstrates that the RGR may play a crucial role in the physiological functions (cell cycle, proliferation, migration, and apoptosis) of keratinocytes. To the best of our knowledge, this is the first report concerning the expression and function of RGR in the human skin.

Previous studies have indicated that the RGR is mainly expressed in the RPE and glial Müller cells of the retina in mammals (Pandey et al., 1994; Shen et al., 1994; Sun et al., 1997) and acts as a mammalian retinaldehyde photoisomerase (Choi et al., 2021). Under light stimulation, the RGR isomerizes retinoids from the all-trans form into the 11-cis form (Jiang et al., 1993). Recently, our team and others have detected some OPNs in normal skin tissues (Tsutsumi et al., 2009; Kim et al., 2013; Haltaufderhyde et al., 2015; de Assis et al., 2018; Regazzetti et al., 2018; Lan et al., 2020; Wang et al., 2020; Lan et al., 2021; Wang et al., 2021). In skin cells including melanocytes, keratinocytes, and fibroblasts, OPNs mediate antiapoptotic effects, melanin synthesis, photoaging, and skin barrier repair following irradiation with UVR or UVA and in other situations (Kim et al., 2013; Lan et al., 2020; Wang et al., 2020; Lan et al., 2021). Whether the light-dependent isomerase reaction is involved in these processes in the human skin is unknown.

To explore the relationship between UV exposure and RGR expression, we collected normal skin tissues of UV-exposed and UV-unexposed skin sites in pairs from the same healthy individuals. Our results showed that a higher expression level of the RGR was detected in the epithelial layer of exposed skin areas, particularly in the basal cell layer and spinous cell layer of the epidermis and cells of the skin appendages. Therefore, we wondered whether the RGR is involved in this photosensitive process in the human skin. We further isolated primary human melanocytes, keratinocytes, and fibroblasts from neonatal foreskin, where we found higher RGR expression in keratinocytes than in melanocytes and fibroblasts. Interestingly, the RGR in keratinocytes was mainly located in the cell membrane and nucleus, which are the same subcellular locations where they are found in human Müller cells of the retina (Shen et al., 1994; Sun et al., 1997; Morshedian et al., 2019).

RGR functions as a retinal photoisomerase in both RPE and Müller glia and may have distinct functional roles (Kiser and Palczewski, 2021). In the RPE, the RGR has been proposed to support visual chromophore production by acting directly as a photoisomerase, stimulating the isomerase activity of the classical visual cycle or eliminating potentially toxic isomers of the retina (Kiser and Palczewski, 2021). In Müller glia, the RGR helps regenerate cone pigments under light and plays a key role in maintaining cone sensitivity (Morshedian et al., 2019). Bovine RGRs are bound to all-trans-retinal in the dark and form a pigment that absorbs blue (λmax = 469 nm) and near-ultraviolet light (λmax = 370 nm) (Hao and Fong, 1996). The chromophore is photoisomerized to 11-cis-retinal. The RGR may specifically bind to all-trans-retinal through its Lys 255 residue (Nickle and Robinson, 2007).

When we exposed keratinocytes to a physiologically relevant dose of UVA (3 J/cm^2^) or UVR (1.5 J/cm^2^) in the presence or absence of all-trans-retinal (Wicks et al., 2011), we did not detect variable RGR expression in keratinocytes after 48 h. Additionally, after we added all-trans-retinal and irradiated the cells with UVA or UVR, RGR expression in the all-trans-retinal + UVA and all-trans-retinal + UVR groups was similar to that in the control group. These results indicate that we did not induce a photoisomerase reaction in keratinocytes by irradiating them with physiologically relevant doses of UVA or UVR.

RGR absorption in the blue and near-UV regions of light depends on pH (Hao and Fong, 1996; Choi et al., 2021). When the pH ranges from 6.5 to 4.0, the absorbance of the RGR in this region of blue light increases noticeably at higher pH (Hao and Fong, 1996). The reason is that the hydrogen ion concentration affects the light absorbance in the near-UV region. Further study is required to detect the light-dependent isomerase reaction in the keratinocytes under the appropriate spectrum and under the relevant hydrogen ion concentrations.

We confirmed that the RGR is expressed in the human skin cell membrane and nucleus and is highly expressed in the cell nucleus. These results are consistent with studies on the retina (Shen et al., 1994; Sun et al., 1997; Morshedian et al., 2019). This subcellular localization of the RGR suggests that the RGR may act as a signaling molecule to mediate the functions of the skin.

Although the RGR is poorly conserved, it sustains the responsiveness of photoreceptor cells (Upton et al., 2021). The mutant RGR causes many diseases, such as dominantly inherited peripapillary choroidal atrophy (c.824dupG, p. I276Nfs*77) (Upton et al., 2021). Abnormal RGR proteins, including RGR-d (an exon-skipping splice variant), strongly inhibit cell growth *in vitro* (Bao et al., 2021). This finding prompted us to investigate the possible modulatory effect of the RGR on cell proliferation in the human skin. Based on the aforementioned studies, we analyzed the RGR expression levels in different skin disease tissues and found that RGR expression in lesions was significantly higher than that in tissues adjacent to the lesions of pathological conditions including psoriasis, seborrheic keratosis, and squamous cell carcinoma. These findings suggest that the RGR may participate in cell proliferation under the aforementioned pathological conditions.

To further investigate the role of the RGR in cell proliferation, we used a small interfering RNA construct to the knockdown RGR in keratinocytes. When the RGR gene was silenced, keratinocytes inhibited cell proliferation and migration and caused cell apoptosis. Three recent studies have reported that OPN family member 3 (OPN3) can induce cell apoptosis in melanocytes (Wang et al., 2020) and hepatocellular carcinoma (Jiao et al., 2012). OPN3 also mediates autophagy in colon cancer (Yoshimoto et al., 2018). In our study, the RGR functioned similar to OPN3. The knockdown of RGR expression reduced the proliferation and migration of the keratinocytes. The cell cycle (S phase) also changed. These findings show that the RGR may be an important protein in the survival of human epidermal keratinocytes, a possibility that deserves further investigation.

In summary, our study is the first to demonstrate RGR expression in skin tissues and their cells including melanocytes, keratinocytes, and fibroblasts and to further explore its subcellular localization. The RGR is mainly expressed in the human skin cell membrane, nucleus, and mitochondria. It is significantly involved in the physiological functions of keratinocytes including the cell cycle, proliferation, migration, and apoptosis. RGR knockdown in human epidermal keratinocytes *in vitro* results in reduced proliferation, migration, and apoptosis. These novel functions of the RGR broaden the putative roles of retinal G-protein-coupled receptors in organisms. Our study provides exciting new evidence about a functional RGR system in keratinocytes and its involvement in physiological and pathological processes. Therefore, we expect that the observed results may indicate the highly relevant function of the RGR in controlling skin cell metabolism and its pharmacological value in skin-related diseases.

## Data Availability

The original contributions presented in the study are included in the article/Supplementary Material, further inquiries can be directed to the corresponding author.

## Supplementary Material

Supplementary Figure S1 RGR regulates the proliferation, migration, and apoptosis of HaCaT cell line. **(A)** Representative HaCaT cell lines after lentivirus transfection with control group, knockdown group, and overexpression group RGR protein by WB assay analysis. β-Tubulin as a loading control. The relative protein levels of RGR and β-tubulin were measured using ImageJ software (n = 3 independent experiments). **p < 0.01, ***p < 0.001. **(B)** Flow cytometry was used to detect the cell cycle. Following lentivirus transfection RGR knockdown, compared with the LV-control-RNAi, lentivirus transfection knockdown RGR HaCaT cell line has a significant decrease in the percentage of S-phase cells at 24 h. Compared with the LV control, lentivirus-transfected RGR overexpression HaCaT cells detected a significant increase in the percentage of S-phase cells at 24 h. *p < 0.05, **p < 0.01. **(C)** Representative wound healing assay demonstrated that the lentivirus transfection RGR knockdown group significantly decreased cell mobility compared with the LV control RNAi group. Compared with the LV control group, the lentivirus transfection RGR overexpression group had increased mobility. Scale bar = 50 μm. **p < 0.01, ***p < 0.001. **(D)** Flow cytometry with annexin V-APC/PI double staining was used to determine apoptotic cells. Following transfection with lentivirus, compared with the LV control RNAi group, the lentivirus-transfected RGR knockdown HaCaT cells had a higher rate of cell apoptosis. Compared to LV controls, lentivirus-transfected RGR overexpression was not significantly different apoptosis rate. ***p < 0.001. **(E)** In the Transwell assay, compared with the LV control RNAi group, lentivirus-transfected RGR knockdown HaCaT cells had significant reduced migration ability. Compared with the LV control group, the lentivirus-transfected overexpression RGR HaCaT cells did not significantly increase migration. Scale = 50 μ m. ***p < 0.001.
