# Supplementary figures and images for: Expression of Retinal G Protein-Coupled Receptor, a Member of the Opsin Family, in Human Skin Cells and Its Mediation of the Cellular Functions of Keratinocytes
Source: Front Cell Dev Biol. 2022 Apr 4;10:787730. doi: 10.3389/fcell.2022.787730 (PMC9014095; doi:10.3389/fcell.2022.787730)

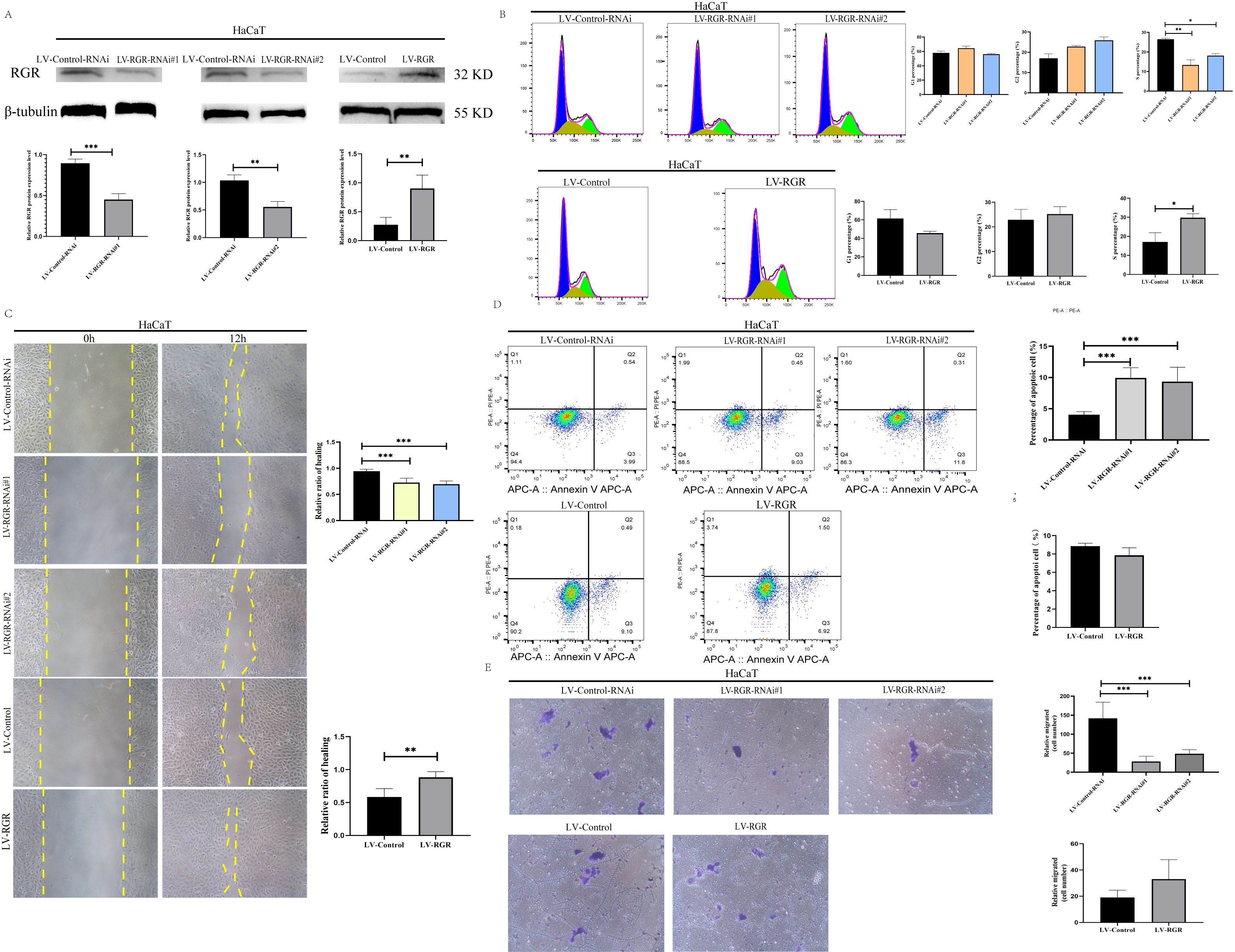

Supplement: Supplementary file 1 [file Image1.TIF]
